# Intracellular Protein Degradation: From a Vague Idea through the Lysosome and the Ubiquitin-Proteasome System and onto Human Diseases and Drug Targeting

**DOI:** 10.5041/RMMJ.10068

**Published:** 2012-01-31

**Authors:** Aaron Ciechanover

**Affiliations:** Nobel Prize Laureate in Chemistry, 2004; Cancer and Vascular Biology Research Center, The Rappaport Faculty of Medicine and Research Institute, Technion-Israel Institute of Technology, Haifa 31096, Israel

**Keywords:** Ubiquitin, proteasome, protein degradation, lysosome, diseases

## Abstract

Between the 1950s and 1980s, scientists were focusing mostly on how the genetic code was transcribed to RNA and translated to proteins, but how proteins were degraded had remained a neglected research area. With the discovery of the lysosome by Christian de Duve it was assumed that cellular proteins are degraded within this organelle. Yet, several independent lines of experimental evidence strongly suggested that intracellular proteolysis was largely non-lysosomal, but the mechanisms involved have remained obscure. The discovery of the ubiquitin-proteasome system resolved the enigma. We now recognize that degradation of intracellular proteins is involved in regulation of a broad array of cellular processes, such as cell cycle and division, regulation of transcription factors, and assurance of the cellular quality control. Not surprisingly, aberrations in the system have been implicated in the pathogenesis of human disease, such as malignancies and neurodegenerative disorders, which led subsequently to an increasing effort to develop mechanism-based drugs.

## INTRODUCTION

The concept of protein turnover is hardly 70 years old. Beforehand, body proteins were viewed as essentially stable constituents that were subject to only minor “wear and tear”: dietary proteins were believed to function primarily as energy-providing fuel, which were independent of the structural and functional proteins of the body. The problem was hard to approach experimentally, as research tools were not available. Important research tools that were lacking at that time were stable isotopes. While radioactive isotopes were developed earlier by George de Hevesy (de Hevesy G. Chemistry 1943. In: *Nobel Lectures in Chemistry 1942–1962*. World Scientific 1999. pp. 5–41), they were mostly unstable and could not be used to follow metabolic pathways. The concept that body structural proteins are static and the dietary proteins are used only as a fuel was challenged by Rudolf Schoenheimer in Columbia University in New York City. Schoenheimer escaped from Germany and joined the Department of Biochemistry in Columbia University founded by Hans T. Clarke.^1^–^3^ There he met Harold Urey who was working in the Department of Chemistry and who discovered deuterium, the heavy isotope of hydrogen, a discovery that enabled him to prepare heavy water, D_2_O. David Rittenberg, who had recently received his PhD in Urey’s laboratory, joined Schoenheimer, and together they entertained the idea of “employing a stable isotope as a label in organic compounds, destined for experiments in intermediary metabolism, which should be biochemically indistinguishable from their natural analog.”^1^ Urey later succeeded in enriching nitrogen with ^15^N, which provided Schoenheimer and Rittenberg with a “tag” for amino acids and, as a result, for the study of protein dynamics. They discovered that following administration of ^15^N-labeled tyrosine to rat only ∼50% can be recovered in the urine, “while most of the remainder is deposited in tissue proteins. An equivalent of protein nitrogen is excreted.”^4^ They further discovered that from the half that was incorporated into body proteins “only a fraction was attached to the original carbon chain, namely to tyrosine, while the bulk was distributed over other nitrogenous groups of the proteins,”^4^ mostly as an αNH_2_ group in other amino acids. These experiments demonstrated unequivocally that the body structural proteins are in a dynamic state of synthesis and degradation, and that even individual amino acids are in a state of dynamic interconversion. Similar results were obtained using ^15^N-labeled leucine.^5^ This series of findings shattered the paradigm in the field at that time that: 1) ingested proteins are completely metabolized and the products are excreted, and 2) that body structural proteins are stable and static. Schoenheimer was invited to deliver the prestigious Edward K. Dunham lecture at Harvard University where he presented his revolutionary findings. After his untimely tragic death in 1941, his lecture notes were edited by Hans Clarke, David Rittenberg, and Sarah Ratner and were published in a small book by Harvard University Press. The editors called the book *The Dynamic State of Body Constituents*,^6^ adopting the title of Schoenheimer’s presentation. In the book, the new hypothesis was clearly presented:

The simile of the combustion engine pictured the steady state flow of fuel into a fixed system, and the conversion of this fuel into waste products. The new results imply that not only the fuel, but the structural materials are in a steady state of flux. The classical picture must thus be replaced by one which takes account of the dynamic state of body structure.^6^

However, the idea that proteins are turning over had not been accepted easily and was challenged as late as the mid-1950s. For example, Hogness and colleagues^7^ studied the kinetics of β-galactosidase in *Escherichia coli* and summarized their findings:

To sum up: there seems to be no conclusive evidence that the protein molecules within the cells of mammalian tissues are in a dynamic state. Moreover, our experiments have shown that the proteins of growing *E. coli* are static. Therefore it seems necessary to conclude that the synthesis and maintenance of proteins within growing cells is not necessarily or inherently associated with a “dynamic state.”^7^

While the experimental study involved the bacterial β-galactosidase, the conclusions were broader, including also the authors’ hypothesis on mammalian proteins. The use of the term “dynamic state” was not incidental, as they challenged directly Schoenheimer’s studies.

Now, after more than seven decades of research in the field of intracellular proteolysis, and with the discovery of the lysosome and later the ubiquitin-proteasome system, it is clear that the field has been revolutionized. We now recognize that intracellular proteins are turning over extensively, that the process is specific, and that the stability of many proteins is regulated individually and can vary under different conditions. From a scavenger, unregulated, and non-specific end process, it has become clear that proteolysis of cellular proteins is a highly complex, temporally controlled, and tightly regulated process that plays major roles in a broad array of basic pathways. Among these processes are cell cycle, development, differentiation, regulation of transcription, antigen presentation, signal transduction, receptor-mediated endocytosis, quality control, and modulation of diverse metabolic pathways. Subsequently, it has changed the paradigm that regulation of cellular processes occurs mostly at the transcriptional and translational levels and has set regulated protein degradation in an equally important position. With the multitude of substrates targeted and processes involved, it has not been surprising to find that aberrations in the pathway have been implicated in the pathogenesis of many diseases, among them certain malignancies, neurodegeneration, and disorders of the immune and inflammatory system. As a result, the system has become a platform for drug targeting, and mechanism-based drugs are currently developed—one of them is already on the market.

## THE LYSOSOME AND INTRACELLULAR PROTEIN DEGRADATION

**Figure 1:**
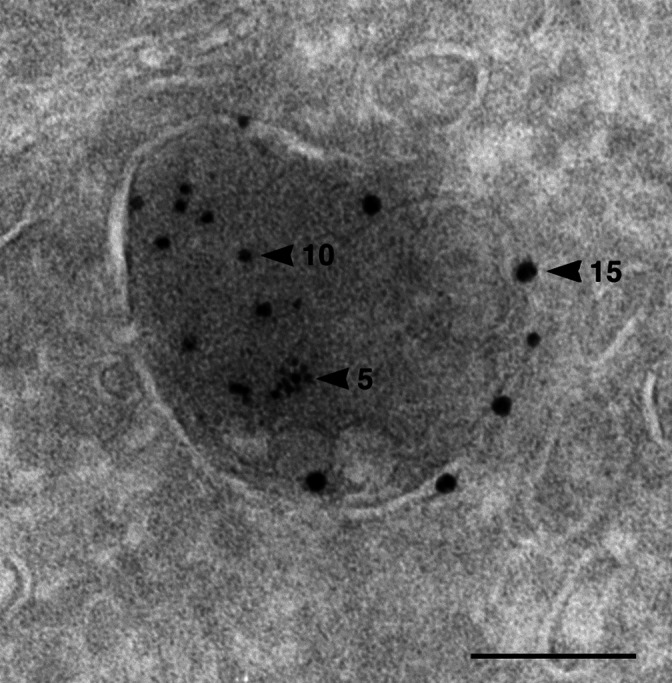
**The lysosome.** Ultrathin cryosection of a rat PC12 cell that had been loaded for 1 hour with bovine serum albumin (BSA)-gold (5-nm particles) and immunolabeled for the lysosomal enzyme cathepsin B (10-nm particles) and the lysosomal membrane protein LAMP1 (15-nm particles). Lysosomes are recognized also by their typical dense content and multiple internal membranes. Bar, 100 nm. Courtesy of Viola Oorschot and Judith Klumperman, Department of Cell Biology, University Medical Centre Utrecht, The Netherlands.

**Figure 2:**
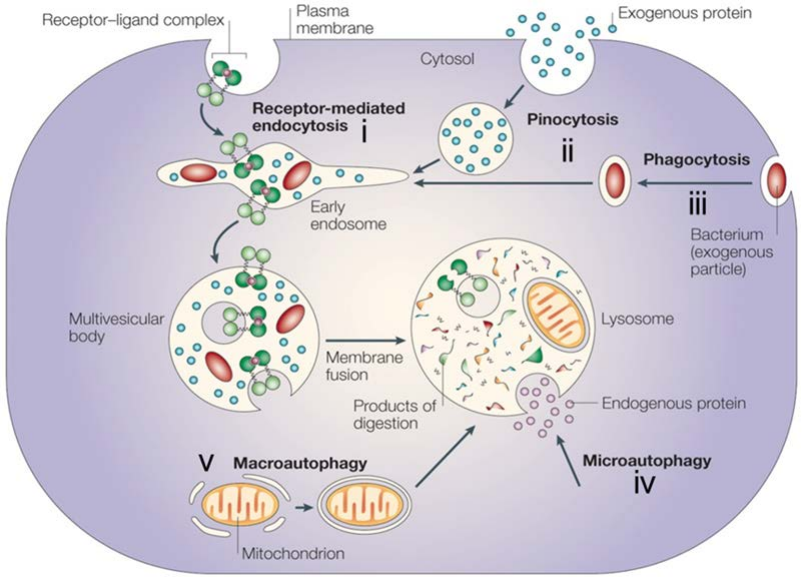
**The four digestive processes mediated by the lysosome (from the upper left corner clockwise).** (i) Specific receptor-mediated endocytosis, (ii) pinocytosis (non-specific engulfment of cytosolic droplets containing extracellular fluid), (iii) phagocytosis (of extracellular particles), and autophagy; (iv) microautophagy of intracellular proteins under basal conditions, and (v) macroautophagy of organelles under stress) (with permission from Nature Publishing Group; published originally in Ciechanover^83^).

In the mid-1950s, Christian de Duve discovered the lysosome (see, for example, de Duve et al.^8^ and Gianetto et al.^9^) (Figure 1). The lysosome was first recognized biochemically in rat liver as a vacuolar structure that contains various hydrolytic enzymes which function optimally at an acidic pH. It is surrounded by a membrane that endows the contained enzymes with the latency that is required to protect the cellular contents from their action (see below). The definition of the lysosome was broadened over the years because it had been recognized that the digestive process is dynamic and involves numerous stages of lysosomal maturation together with the digestion of both exogenous proteins (which are targeted to the lysosome through receptor-mediated endocytosis and pinocytosis) and exogenous particles (which are targeted via phagocytosis; the two processes are known as heterophagy), as well as digestion of endogenous proteins and cellular organelles (which are targeted by micro- and macroautophagy; see Figure 2). The lysosomal/vacuolar system as we currently recognize it is a discontinuous and heterogeneous digestive system that also includes structures that are devoid of hydrolases—for example, early endosomes which contain endocytosed receptor–ligand complexes and pinocytosed/phagocytosed extracellular contents. At the other extreme it includes the residual bodies—the end products of the completed digestive processes of heterophagy and autophagy. In between these extremes one can observe: primary/nascent lysosomes that have not been engaged yet in any proteolytic process; early autophagic vacuoles that might contain intracellular organelles; intermediate/late endosomes and phagocytic vacuoles (heterophagic vacuoles) that contain extracellular contents/particles; and multivesicular bodies (MVBs) which are the transition vacuoles between endosomes/phagocytic vacuoles and the digestive lysosomes.

The discovery of the lysosome, along with independent experiments that were carried out at the same time and that have further strengthened the notion that cellular proteins are indeed in a constant state of synthesis and degradation (see, for example, Simpson^10^), led scientists to feel, for the first time, that they have at hand an organelle that can potentially mediate degradation of intracellular proteins. The fact that the proteases were separated from their substrates by a membrane provided an explanation for controlled degradation, and the only problem left to be explained was how the substrates are translocated into the lysosomal lumen, exposed to the activity of the lysosomal proteases, and degraded. An important discovery in this respect was the unraveling of the basic mechanism of action of the lysosome—autophagy (reviewed in Mortimore et al.^11^). Under basal metabolic conditions, portions of the cytoplasm, which contain the entire cohort of cellular proteins, are segregated within a membrane-bound compartment and are then fused to a primary nascent lysosome and their contents digested. This process was called microautophagy. Under more extreme conditions, starvation for example, mitochondria, endoplasmic reticulum membranes, glycogen bodies, and other cytoplasmic entities can also be engulfed by a process called macroautophagy (see, for example, Ashford et al.^12^; the different modes of action of the lysosome in digesting extra- and intracellular proteins are shown in Figure 2).

However, over a period of more than two decades, between the mid-1950s and the late 1970s, it has become gradually more and more difficult to explain several aspects of intracellular protein degradation based on the known mechanisms of lysosomal activity: accumulating lines of independent experimental evidence indicated that the degradation of at least certain classes of cellular proteins must be non-lysosomal. Yet, in the absence of any “alternative,” researchers came up with different explanations, some more substantiated and others less, to defend the “lysosomal” hypothesis.

First was the gradual discovery, from different laboratories, that different proteins vary in their stabilities and their half-life times can span three orders of magnitude, from a few minutes to many days. Thus, the t_1/2_ of ornithine decarboxylase (ODC) is ∼10 min, while that of glucose-6-phosphate dehydrogenase (G6PD) is 15 hours (for review articles, see, for example, Schimke et al.^13^ and Goldberg et al.^14^). Also, rates of degradation of many proteins were shown to change with changing physiological conditions, such as availability of nutrients or hormones. It was conceptually difficult to reconcile the findings of distinct and changing half-lives of different proteins with the mechanism of action of the lysosome, where the microautophagic vesicle contains the entire cohort of cellular (cytosolic) proteins that are therefore expected to degrade at the same rate. Similarly, changing pathophysiological conditions, such as starvation or resupplementation of nutrients, were expected to affect the stability of all cellular proteins to the same extent. Clearly, this was not the case.

Another source of concern about the lysosome as the organelle in which intracellular proteins are degraded was the finding that specific and general inhibitors of lysosomal proteases have different effects on different populations of proteins, making it clear that distinct classes of proteins are targeted by different proteolytic machineries. Thus, the degradation of endocytosed/pinocytosed extracellular proteins was significantly inhibited, a partial effect was observed on the degradation of long-lived cellular proteins, and almost no effect was detected on the degradation of short-lived and abnormal/mutated proteins.

Finally, the thermodynamically paradoxical observation that the degradation of cellular proteins requires metabolic energy and, more importantly, the emerging evidence that the proteolytic machinery uses the energy directly were in contrast with the known mode of action of lysosomal proteases that under the appropriate acidic conditions, and similar to all known proteases, degrade proteins in an exergonic manner.

The assumption that the degradation of intracellular proteins is mediated by the lysosome was nevertheless logical. Proteolysis results from direct interaction between the target substrates and proteases, and therefore it was clear that active proteases cannot be free in the cytosol which would have resulted in destruction of the cell. Thus, it was recognized that any suggested proteolytic machinery that mediates intracellular protein degradation must also be equipped with a mechanism that separates—physically or virtually—the proteases and their substrates and enables them to associate only when needed. The lysosomal membrane provided this fencing mechanism. Obviously, nobody could have predicted that a new mode of post-translational modification—ubiquitination—could function as a proteolysis signal and that untagged proteins would remain protected. Thus, while the structure of the lysosome could explain the separation necessary between the proteases and their substrates, and autophagy could explain the mechanism of entry of cytosolic proteins into the lysosomal lumen, major problems have remained unsolved. Important among them were: 1) the varying half-lives, 2) the energy requirement, and 3) the distinct response of different populations of proteins to lysosomal inhibitors. Thus, according to one model, it was proposed that different proteins have different sensitivities to lysosomal proteases, and their half-lives *in vivo* correlate with their sensitivity to the action of lysosomal proteases *in vitro*.^15^ To explain an extremely long half-life of a protein that was nevertheless sensitive to lysosomal proteases, or alterations in the stability of a single protein under various physiological states, it was suggested that, although all cellular proteins are engulfed into the lysosome, only the short-lived proteins are degraded, whereas the long-lived proteins exit back into the cytosol:

To account for differences in half-life among cell components or of a single component in various physiological states, it was necessary to include in the model the possibility of an exit of native components back to the extralysosomal compartment.^16^

According to a different model, selectivity was determined by the binding affinity of the different proteins to the lysosomal membrane which controls their entry rates into the lysosome and subsequently their degradation rates.^17^ For a selected group of proteins, such as the gluconeogenetic enzymes phosphoenol-pyruvate carboxykinase (PEPCK) and fructose-1,6-biphosphatase, it was suggested, though not firmly substantiated, that their degradation in the yeast vacuole was regulated by glucose via a mechanism called “catabolite inactivation” that possibly involves their phosphorylation. However, this regulated mechanism for vacuolar degradation was limited only to a small and specific group of proteins (see for example Müller et al.^18^; reviewed in Holzer^19^). More recent studies have shown that at least for stress-induced macroautophagy, a general sequence of amino acids, KFFERQ, directs, via binding to a specific “receptor” and along with cytosolic and lysosomal chaperones, the regulated entry of many cytosolic proteins into the lysosomal lumen. While further corroboration of this hypothesis is still required, it can only explain the mass entry of a large population of proteins that contain a homologous sequence, but not the targeting for degradation of a specific protein under defined conditions (reviewed in Majeski et al.^20^ and Cuervo et al.^21^). The energy requirement for protein degradation was described as indirect, and necessary, for example, for protein transport across the lysosomal membrane^22^ and/or for the activity of the H^+^ pump and the maintenance of the low acidic intralysosomal pH that is necessary for optimal activity of the proteases.^23^ We now know that both mechanisms require energy. In the absence of any alternative, and with lysosomal degradation as the most logical explanation for targeting all known classes of proteins at the time, Christian de Duve summarized his view on the subject in a review article published in the mid-1960s, saying: “Just as extracellular digestion is successfully carried out by the concerted action of enzymes with limited individual capacities, so, we believe, is intracellular digestion.”^24^ The problem of different sensitivities of distinct protein groups to lysosomal inhibitors has remained unsolved and may have served as an important trigger in the future quest for a non-lysosomal proteolytic system.

Progress in identifying the elusive, non-lysosomal proteolytic system(s) was hampered by the lack of a cell-free preparation that could faithfully replicate the cellular proteolytic events—i.e. degrading proteins in a specific and energy-requiring mode. An important breakthrough was made by Rabinovitz and Fisher who found that rabbit reticulocytes degrade abnormal, amino acid analog-containing hemoglobin.^25^ Their experiments modeled known disease states, the hemoglobinopathies. In these diseases abnormal mutated hemoglobin chains (such as sickle cell hemoglobin) or excess of unassembled normal hemoglobin chains (which are synthesized normally, but also excessively in thalassemias, diseases in which the pairing chain is not synthesized at all or is mutated and rapidly degraded, and consequently the bi-heterodimeric hemoglobin complex is not assembled) are rapidly degraded in the reticulocyte.^26^,^27^ Reticulocytes are terminally differentiating red blood cells that do not contain lysosomes. Therefore, it was postulated that the degradation of hemoglobin in these cells was mediated by a non-lysosomal machinery. Etlinger and Goldberg^28^ were the first to isolate and characterize a cell-free proteolytic preparation from reticulocytes. The crude extract selectively degraded abnormal hemoglobin, required ATP hydrolysis, and acted optimally at a neutral pH, which further corroborated the assumption that the proteolytic activity was of a non-lysosomal origin. A similar system was isolated and characterized later by Hershko et al.^29^ Additional studies by this group led subsequently to resolution, characterization, and purification of the major enzymatic components from this extract and to the discovery of the ubiquitin signaling system (see below).

## THE LYSOSOME HYPOTHESIS IS CHALLENGED

As mentioned above, the unraveled mechanism(s) of action of the lysosome could explain only partially, and at times not satisfactorily, several key emerging characteristics of intracellular protein degradation. Among them were the heterogeneous stability of individual proteins, the effect of nutrients and hormones on their degradation, and the dependence of intracellular proteolysis on metabolic energy. The differential effect of selective inhibitors on the degradation of different classes of cellular proteins (see above but mostly below) could not be explained at all.

The evolution of methods to monitor protein kinetics in cells, together with the development of specific and general lysosomal inhibitors, has resulted in the identification of different classes of cellular proteins, long- and short-lived, and the discovery of the differential effects of the inhibitors on these groups (see, for example, Knowles et al.^30^ and Neff et al.^31^). An elegant experiment in this respect was carried out by Brian Poole and his colleagues in the Rockefeller University. Poole was studying the effect of lysosomotropic agents, weak bases such as ammonium chloride and chloroquine, which accumulate in the lysosome and dissipate its low acidic pH. It was assumed that this mechanism underlies also the anti-malarial activity of chloroquine and similar drugs where they inhibit the activity of the parasite’s lysosome, “paralyzing” its ability to digest the host’s hemoglobin during the intra-erythrocytic stage of its life cycle. Poole and his colleagues metabolically labeled endogenous proteins in living macrophages with ^3^H-leucine and “fed” them with dead macrophages that had been previously labeled with ^14^C-leucine. They assumed, apparently correctly, that the dead macrophage debris and proteins will be phagocytosed by live macrophages and targeted to the lysosome for degradation. They monitored the effect of lysosomotropic agents on the degradation of these two protein populations; in particular, they studied the effect of the weak bases chloroquine and ammonium chloride (which enter the lysosome and neutralize the H^+^ ions) and the acid ionophore X537A which dissipates the H^+^ gradient across the lysosomal membrane. They found that these drugs specifically inhibited the degradation of extracellular proteins but not that of intracellular proteins.^32^ Poole summarized these experiments and explicitly predicted the existence of a non-lysosomal proteolytic system that degrades intracellular proteins:

Some of the macrophages labeled with tritium were permitted to endocytise the dead macrophages labeled with ^14^C. The cells were then washed and replaced in fresh medium. In this way we were able to measure in the same cells the digestion of macrophage proteins from two sources. The exogenous proteins will be broken down in the lysosomes, while the endogenous proteins will be broken down wherever it is that endogenous proteins are broken down during protein turnover.^33^

The requirement for metabolic energy for the degradation of both prokaryotic^34^ and eukaryotic^10^,^35^ proteins was difficult to explain. Proteolysis is an exergonic process, and the thermodynamically paradoxical energy requirement for intracellular proteolysis made researchers believe that energy cannot be consumed directly by proteases or the proteolytic process per se and is used indirectly. As Simpson summarized his findings:^10^

The data can also be interpreted by postulating that the release of amino acids from protein is itself directly dependent on energy supply. A somewhat similar hypothesis, based on studies on autolysis in tissue minces, has recently been advanced, but the supporting data are very difficult to interpret. However, the fact that protein hydrolysis as catalyzed by the familiar proteases and peptidases occurs exergonically, together with the consideration that autolysis in excised organs or tissue minces continues for weeks, long after phosphorylation or oxidation ceased, renders improbable the hypothesis of the direct energy dependence of the reactions leading to protein breakdown.^10^

Being cautious, however, and probably unsure about this unequivocal conclusion, Simpson still left a narrow orifice opened for a proteolytic process that requires energy in a direct manner: “However, the results do not exclude the existence of two (or more) mechanisms of protein breakdown, one hydrolytic, the other energy-requiring.”^10^ Since any proteolytic process must be at one point or another hydrolytic, the statement that makes a distinction between a hydrolytic process and an energy-requiring yet non-hydrolytic process is not clear. Judging the statement from an historical point of view and knowing the mechanism of action of the ubiquitin system, where energy is required also in the pre-hydrolytic step (ubiquitin conjugation), Simpson may have thought of a two-step mechanism but did not give it a clear description. At the end of this clearly understandable but at the same time difficult and convoluted deliberation, Simpson left us with a vague explanation linking protein degradation to protein synthesis, a process that was known to require metabolic energy:

The fact that a supply of energy seems to be necessary for both the incorporation and the release of amino acids from protein might well mean that the two processes are interrelated. Additional data suggestive of such a view are available from other types of experiments. Early investigations on nitrogen balance by Benedict, Folin, Gamble, Smith, and others point to the fact that the rate of protein catabolism varies with the dietary protein level. Since the protein level of the diet would be expected to exert a direct influence on synthesis rather than breakdown, the altered catabolic rate could well be caused by a change in the rate of synthesis.^10^

With the discovery of lysosomes in eukaryotic cells it could be argued that energy was required for the transport of substrates into the lysosome or for maintenance of the low intralysosomal pH (see above), for example. The observation by Hershko and Tomkins that the activity of tyrosine aminotransferase (TAT) was stabilized following depletion of ATP^36^ indicated that energy could be required at an early stage of the proteolytic process, most probably before proteolysis occurs. Yet, it did not provide a clue to the mechanism involved: energy could be used, for example, for specific modification of TAT, e.g. phosphorylation, that would sensitize it to degradation by the lysosome or by a yet unknown proteolytic mechanism, or for a modification that activates its putative protease. It could also be used for a more general lysosomal mechanism—one that involves transport of TAT into the lysosome, for example. The energy inhibitors inhibited almost completely degradation of the entire population of cell proteins, confirming previous studies (e.g. Simpson^10^) and suggesting a general role for energy in protein catabolism. Yet, an interesting finding was that energy inhibitors had an effect that was distinct from that of protein synthesis inhibitors which affected only enhanced degradation (induced by steroid hormone depletion) but not basal degradation. This finding ruled out, at least partially, a tight linkage between protein synthesis and degradation. In bacteria, which lack lysosomes, an argument involving energy requirement for lysosomal degradation could not have been proposed, but other indirect effects of ATP hydrolysis could have affected proteolysis in *E. coli*, such as phosphorylation of substrates and/or proteolytic enzymes, or maintenance of the “energized membrane state.” According to this model, proteins could become susceptible to proteolysis by changing their conformation, for example, following association with the cell membrane that maintains a local, energy-dependent gradient of a certain ion. While such an effect was ruled out,^37^ and since there was no evidence for a phosphorylation mechanism (although the proteolytic machinery in prokaryotes had not been identified at that time), it seemed that, at least in bacteria, energy was required directly for the proteolytic process. In any event, the requirement for metabolic energy for protein degradation in both prokaryotes and eukaryotes, a process that is exergonic thermodynamically, strongly indicated that in cells proteolysis is highly regulated and that a similar principle/mechanism has been preserved along evolution of the two kingdoms. From the possible direct requirement for ATP in degradation of proteins in bacteria, it was not too unlikely to assume a similar direct mechanism in the degradation of cellular proteins in eukaryotes. Supporting this notion was the description of the cell-free proteolytic system in reticulocytes,^28^,^29^ a cell that lacks lysosomes, which indicates that energy is probably required directly for the proteolytic process, although here, too, the underlying mechanisms had remained enigmatic at the time. Yet, the description of the cell-free system paved the road for detailed dissection of the underlying mechanisms involved.

## THE UBIQUITIN-PROTEASOME SYSTEM

**Figure 3:**
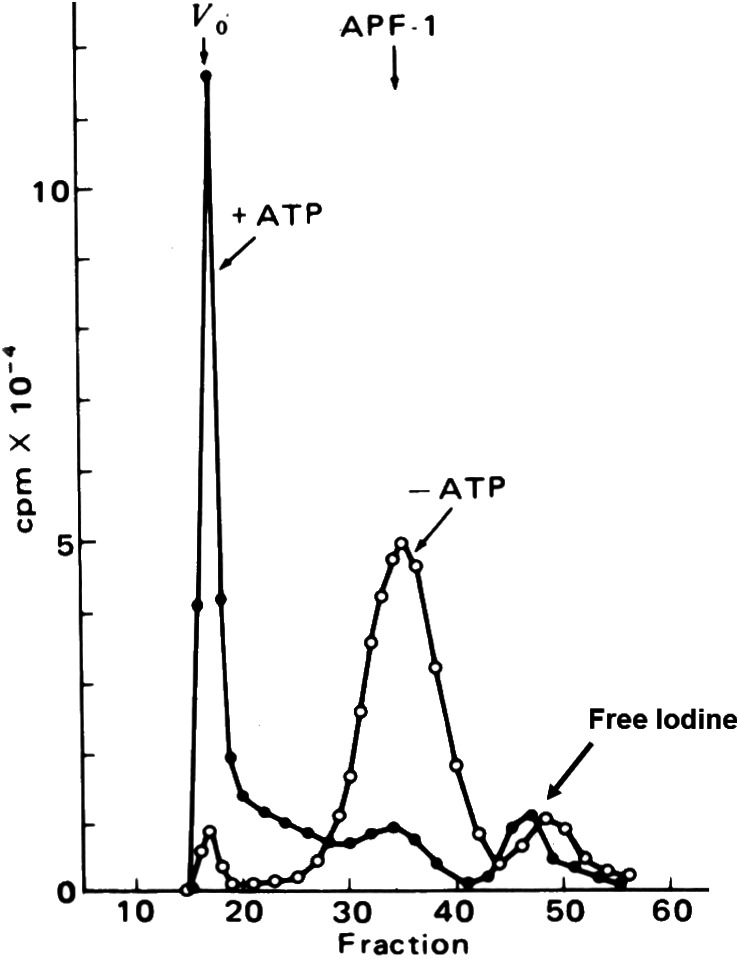
**APF-1/ubiquitin is shifted to high-molecular-mass compound(s) following incubation in ATP-containing crude cell extract.** ^125^I-labeled APF-1/ubiquitin was incubated with reticulocyte crude fraction II in the absence (open circles) or presence (closed circles) of ATP, and the reaction mixtures were resolved via gel filtration chromatography. Shown is the radioactivity measured in each fraction. As can be seen, following addition of ATP, APF-1/ubiquitin becomes covalently attached to some component(s) in fraction II, which could be another enzyme of the system or its substrate(s) (with permission from Proceedings of the National Academy of the USA; published originally in Ciechanover et al.^39^).

**Figure 4:**
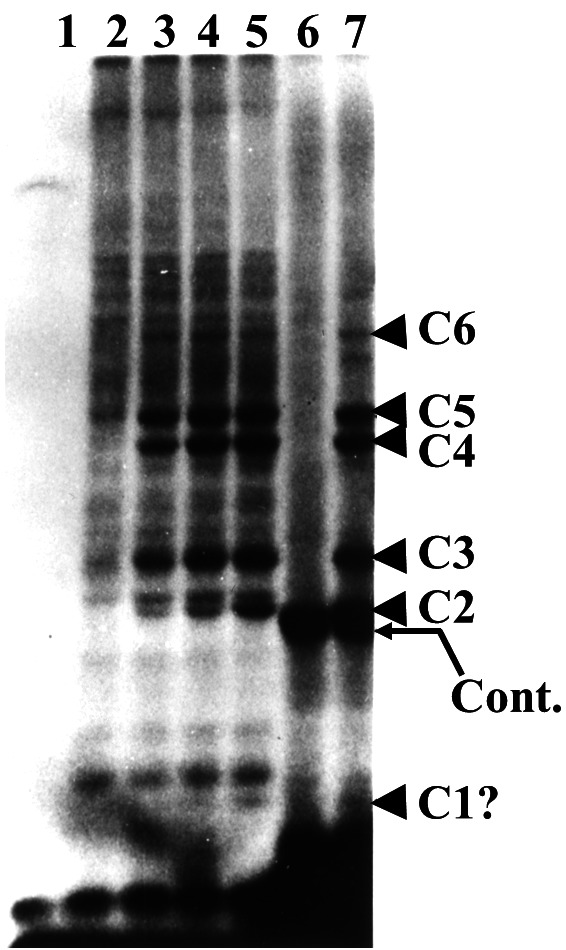
**Multiple molecules of APF-1/ubiquitin are conjugated to the proteolytic substrate, probably signaling it for degradation.** To interpret the data described in the experiment depicted in Figure 2 and to test the hypothesis that APF-1 is conjugated to the target proteolytic substrate, ^125^I-APF-1/ubiquitin was incubated along with crude fraction II (Figure 3 and text) in the absence (lane 1) or presence (lanes 2–5) of ATP and in the absence (lanes 1 and 2) or presence (lanes 3–5) of increasing concentrations of unlabeled lysozyme. Reaction mixtures resolved in lanes 6 and 7 were incubated in the absence (lane 6) or presence (lane 7) of ATP, and included unlabeled APF-1/ubiquitin and ^125^I-labeled lysozyme. C1–C6 denote specific APF-1/ubiquitin-lysozyme adducts in which the number of APF-1/ubiquitin moieties bound to the lysozyme moiety of the adduct is increasing, probably from 1 to 6. Reaction mixtures were resolved via sodium dodecyl sulfate-polyacrylamide gel electrophoresis (SDS-PAGE) and visualized following exposure to an X-ray film (autoradiography) (with permission from Proceedings of the National Academy of the USA; published originally in Hershko et al.^40^).

The cell-free proteolytic system from reticulocytes^28^,^29^ turned out to be an important and rich source for the purification and characterization of the enzymes that are involved in the ubiquitin-proteasome system. Initial fractionation of the crude reticulocyte cell extract on the anion exchange resin diethylaminoethyl cellulose yielded two fractions which were both required to reconstitute the energy-dependent proteolytic activity that is found in the crude extract: the unadsorbed, flow-through material was denoted fraction I, and the high-salt eluate of the adsorbed proteins was denoted fraction II (Table 1).^38^ This was an important observation and a lesson for the future dissection of the system. For one it suggested that the system was not composed of a single “classical” protease that has evolved evolutionarily to acquire energy dependence (although such energy-dependent proteases, the mammalian 26S proteasome (see below) and the prokaryotic *Lon* gene product have been described later) but that it was made of at least two components. This finding of a two-component, energy-dependent protease left the researchers with no paradigm to follow, and, in attempts to explain the finding, they suggested, for example, that the two fractions could represent an inhibited protease and its activator. Second, learning from this reconstitution experiment and the essential dependence between the two active components, we continued to reconstitute activity from resolved fractions whenever we encountered a loss of activity along further purification steps. This biochemical “complementation” approach resulted in the discovery of additional enzymes of the system, all required to be present in the reaction mixture in order to catalyze the multistep proteolysis of the target substrate. We chose first to purify the active component from fraction I. It was found to be a small, ∼8.5 kDa, heat-stable protein that was designated ATP-dependent proteolysis factor 1 (APF-1). APF-1 was later identified as ubiquitin (see below; I am using the term APF-1 to the point where it was identified as ubiquitin and then changing terminology accordingly). In retrospect, the decision to start the purification efforts with fraction I turned out to be important, as fraction I contained only one single protein—APF-1—that was necessary to stimulate proteolysis of the model substrate we used at the time, while fraction II turned out to contain many more. Later studies showed that fraction I contains other components necessary for the degradation of other substrates, but these were not necessary for the reconstitution of the system at that time. This enabled us not only to purify APF-1 but also to decipher quickly its mode of action. If we had started our purification efforts with fraction II, we would have encountered a significantly bumpier road. A critically important finding that paved the way for future developments in the field was that multiple moieties of APF-1 are covalently conjugated to the target substrate when incubated in the presence of fraction II, and the modification requires ATP (Figure 3 and Figure 4).^39^,^40^ It was also found that the modification is reversible and APF-1 could be removed from the substrate or its degradation products.^40^

**Table 1:** Resolution of the ATP-dependent proteolytic activity from crude reticulocyte extract into two essentially required complementing activities (adapted from Ciechanover et al.^38^; with permission from Elsevier/Biochem Biophys Res Commun).

**Fraction**	**Degradation of [^3^H]globin (%)**	

	**–ATP**	**+ATP**
Lysate	1.5	10
Fraction I	0.0	0.0
Fraction II	1.5	2.7
Fraction I and Fraction II	1.6	10.6

The discovery that APF-1 was covalently conjugated to protein substrates and stimulates their proteolysis in the presence of ATP and crude fraction II led in 1980 to the proposal of a model according to which protein substrate modification by multiple moieties of APF-1 targets it for degradation by a downstream, at that time yet unidentified, protease that cannot recognize the unmodified substrate; following degradation, reusable APF-1 was released.^40^ Amino acid analysis of APF-1, along with its known molecular mass and other general characteristics, raised the suspicion that APF-1 was ubiquitin,^41^ a known protein of previously unknown function. Indeed, Wilkinson and colleagues confirmed unequivocally that APF-1 was indeed ubiquitin.^42^ Ubiquitin had been first described as a small, heat-stable, and highly evolutionarily conserved protein of 76 residues. It was first purified during the isolation of thymopoietin^43^ and was subsequently found to be ubiquitously expressed in all kingdoms of living cells, including prokaryotes.^44^ Interestingly, it was initially found to have lymphocyte-differentiating properties, a characteristic that was attributed to the stimulation of adenylate cyclase.^44^,^45^ Accordingly, it was named UBIP for ubiquitous immunopoietic polypeptide.^44^ However, later studies showed that ubiquitin was not involved in the immune response^46^ and that it was a contaminating endotoxin in the preparation that generated the adenylate cyclase and the T-cell differentiating activities. Furthermore, the sequence of several eubacteria and archaebacteria genomes as well as biochemical analyses in these organisms (unpublished) showed that ubiquitin was restricted only to eukaryotes. The finding of ubiquitin in bacteria^44^ was probably due to contamination of the bacterial extract with yeast ubiquitin derived from the yeast extract in which the bacteria were grown. While in retrospect the name ubiquitin is a misnomer, as it is restricted to eukaryotes and is not ubiquitous as was previously thought, it has remained the name of the protein. The reason is probably because it was the name that was first assigned to the protein, and scientists and nomenclature committees tend, in general, to respect this tradition. Accordingly, and in order to avoid confusion, I suggest that the names of other novel enzymes and components of the ubiquitin system, but also of other systems as well, should remain as first coined by their discoverers.

An important development in the ubiquitin research field was the discovery that a single ubiquitin moiety can be covalently conjugated to histones, particularly to histones H2A and H2B. While the function of these adducts has remained elusive until recently, their structure was unraveled in the mid-1970s. The structure of the ubiquitin conjugate of H2A (uH2A; also designated protein A24) was deciphered by Goldknopf and Busch^47^,^48^ and by Hunt and Dayhoff^49^ who found that the two proteins are linked through a fork-like, branched isopeptide bond between the carboxy-terminal glycine of ubiquitin (Gly–76) and the ε-NH_2_ group of an internal lysine (Lys–119) of the histone molecule. The isopeptide bond found in the histone-ubiquitin adduct was suggested to be identical to the bond that was found between ubiquitin and the target proteolytic substrate^50^ and between the ubiquitin moieties in the polyubiquitin chain^51^,^52^ that was synthesized on the substrate and that functions as a proteolysis recognition signal for the downstream 26S proteasome. In this particular polyubiquitin chain the linkage is between Gly–76 of one ubiquitin moiety and internal Lys–48 of the previously conjugated moiety. Only Lys–48-based ubiquitin chains are recognized by the 26S proteasome and serve as proteolytic signals. In recent years it has been shown that the first ubiquitin moiety can also be attached in a linear mode to the N-terminal residue of the proteolytic target substrate.^53^ However, the subsequent ubiquitin moieties are generating Lys–48-based polyubiquitin chain on the first linearly fused moiety. N-terminal ubiquitination is clearly required for targeting naturally occurring lysine-less proteins for degradation. Yet, several lysine-containing proteins have also been described that traverse this pathway—the muscle-specific transcription factor MyoD, for example. In these proteins the internal lysine residues are probably not accessible to the cognate ligases. Other types of polyubiquitin chains have also been described that are not involved in targeting the conjugated substrates for proteolysis. Thus, a Lys–63-based polyubiquitin chain has been described that is probably necessary to activate transcription factors (reviewed recently in Muratani et al.^54^). Interestingly, the role of monoubiquitination of histones has also been identified recently, and this modification is also involved in regulation of transcription, probably via modulation of the structure of the nucleosomes (for recent reviews, see, for example, Zhang^55^ and Osley^56^).

The identification of APF-1 as ubiquitin, and the discovery that a high-energy isopeptide bond, similar to the one that links ubiquitin to histone H2A, links it also to the target proteolytic substrate, resolved at that time the enigma of the energy requirement for intracellular proteolysis (see, however, below) and paved the road to the untangling of the complex mechanism of isopeptide bond formation. This process turned out to be similar to that of peptide bond formation that is catalyzed by tRNA synthetase following amino acid activation during protein synthesis or during the non-ribosomal synthesis of short peptides.^57^ Using the unraveled mechanism of ubiquitin activation and immobilized ubiquitin as a “covalent” affinity bait, the three enzymes that are involved in the cascade reaction of ubiquitin conjugation were purified by Ciechanover, Hershko, and their colleagues. These enzymes are: 1) E1, the ubiquitin-activating enzyme, 2) E2, the ubiquitin-carrier protein, and 3) E3, the ubiquitin-protein ligase.^58^,^59^ The discovery of an E3, which was a specific substrate-binding component, indicated a possible solution to the problem of the varying stabilities of different proteins—they might be specifically recognized and targeted by different ligases.

In a short period, the ubiquitin-tagging hypothesis received substantial support. For example, Chin and colleagues injected into HeLa cells labeled ubiquitin and hemoglobin and denatured the injected hemoglobin by oxidizing it with phenylhydrazine. They found that ubiquitin conjugation to globin was markedly enhanced by denaturation of hemoglobin and the concentration of globin-ubiquitin conjugates was proportional to the rate of hemoglobin degradation.^60^ Hershko and colleagues observed a similar correlation for abnormal, amino acid analog-containing short-lived proteins.^61^ A previously isolated cell cycle arrest mutant that loses the ubiquitin-histone H2A adduct at the permissive temperature^62^ was found by Finley et al. to harbor a thermolabile E1.^63^ Following heat inactivation, the cells fail to degrade normal short-lived proteins.^64^ Although the cells did not provide direct evidence for substrate ubiquitination as a destruction signal, they still provided the strongest direct linkage between ubiquitin conjugation and degradation.

At this point, the only missing link was the identification of the downstream protease that would specifically recognize ubiquitinated substrates. Tanaka and colleagues identified a second ATP-requiring step in the reticulocyte proteolytic system, which occurred after ubiquitin conjugation,^65^ and Hershko and colleagues demonstrated that the energy was required for conjugate degradation.^66^ An important advance in the field was a discovery by Hough and colleagues, who partially purified and characterized a high-molecular-mass alkaline protease that degraded ubiquitin adducts of lysozyme, but not untagged lysozyme, in an ATP-dependent mode.^67^ This protease, which was later called the 26S proteasome (see below), provided all the necessary criteria for being the specific proteolytic arm of the ubiquitin system. This finding was confirmed, and the protease was further characterized by Waxman and colleagues who found that it was an unusually large, ∼1.5 MDa, enzyme, unlike any other known protease.^68^ A further advance in the field was the discovery^69^ that a smaller neutral multi-subunit 20S protease complex that was discovered together with the larger 26S complex was similar to a “multicatalytic proteinase complex” (MCP) that had been described earlier in bovine pituitary gland by Wilk and Orlowski.^70^ This 20S protease was ATP-independent and has different catalytic activities, cleaving on the carboxy-terminal side of hydrophobic, basic, and acidic residues. Hough and colleagues raised the possibility—although they did not show it experimentally—that this 20S protease could be a part of the larger 26S protease that degrades the ubiquitin adducts.^69^ Later studies showed that, indeed, the 20S complex is the core catalytic particle of the larger 26S complex.^71^,^72^ However, strong evidence that the active “mushroom”-shaped 26S protease was generated through the assembly of two distinct subcomplexes—the catalytic 20S cylinder-like MCP and an additional 19S ball-shaped subcomplex (that was predicted to have a regulatory role)—was provided only in the early 1990s by Hoffman and colleagues^73^ who mixed the two purified particles and generated the active 26S enzyme.

**Figure 5:**
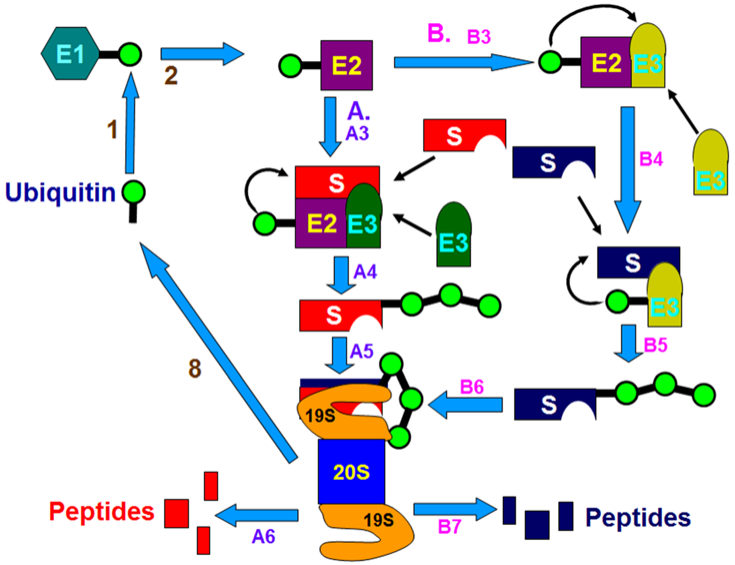
**The ubiquitin-proteasome proteolytic system.** Ubiquitin is activated by the ubiquitin-activating enzyme, E1 (1) followed by its transfer to a ubiquitin-carrier protein (ubiquitin-conjugating enzyme, UBC), E2 (2). E2 transfers the activated ubiquitin moieties to the protein substrate that is bound specifically to a unique ubiquitin ligase E3 (A and B). In the case of RING finger ligases, the transfer is direct (A3). Successive conjugation of ubiquitin moieties to one another generates a polyubiquitin chain (A4) that serves as the binding (A5) signal for the downstream 26S proteasome that degrades the target substrates to peptides (A6). In the case of HECT domain ligases, ubiquitin generates an additional thiol-ester intermediate on the ligase (B3) and only then is transferred to the substrate (B4). Successive conjugation of ubiquitin moieties to one another generates a polyubiquitin chain (B5) that binds to the 26S proteasome (B6) followed by degradation of the substrate to peptides (B7). Free and reusable ubiquitin is released by de-ubiquitinating enzymes (DUBs) (8).

**Figure 6:**
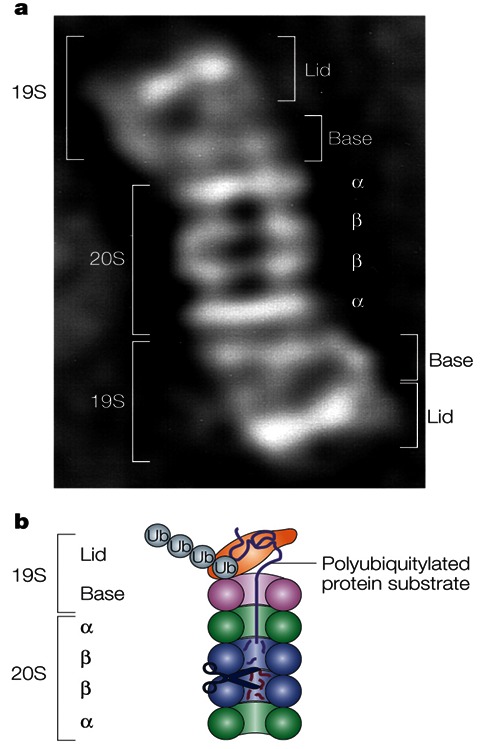
**The proteasome.** The proteasome is a large, 26S, multicatalytic protease that degrades polyubiquitinated proteins to small peptides. It is composed of two subcomplexes: a 20S core particle (CP) that carries the catalytic activity and a regulatory 19S regulatory particle (RP). The 20S CP is a barrel-shaped structure composed of four stacked rings, two identical outer α rings and two identical inner β rings. The eukaryotic α and β rings are composed each of seven distinct subunits, giving the 20S complex the general structure of α1–7β1–7β1–7α1–7. The catalytic sites are localized to some of the β subunits. Each extremity of the 20S barrel can be capped by a 19S RP, each composed of 17 distinct subunits: 9 in a “base” subcomplex, and 8 in a “lid” subcomplex. One important function of the 19S RP is to recognize ubiquitinated proteins and other potential substrates of the proteasome. Several ubiquitin-binding subunits of the 19S RP have been identified; however, their biological roles or modes of action have not been discerned. A second function of the 19S RP is to open an orifice in the α ring that will allow entry of the substrate into the proteolytic chamber. Also, since a folded protein would not be able to fit through the narrow proteasomal channel, it is assumed that the 19S particle unfolds substrates and inserts them into the 20S CP. Both the channel opening function and the unfolding of the substrate require metabolic energy, and, indeed, the 19S RP “base” contains six different ATPase subunits. Following degradation of the substrate, short peptides derived from the substrate are released, as well as reusable ubiquitin. **a:** Electron microscopy image of the 26S proteasome from the yeast S. cerevisiae. **b:** Schematic representation of the structure and function of the 26SA proteasome (with permission from Nature Publishing Group; published originally in Ciechanover et al.^83^).

The proteasome is a large, 26S, multicatalytic protease that degrades polyubiquitinated proteins to small peptides. It is composed of two subcomplexes: a 20S core particle (CP), that carries the catalytic activity, and a 19S regulatory particle (RP). The 20S CP is a barrel-shaped structure composed of four stacked rings, two identical outer β rings and two identical inner β rings. The eukaryotic α and β rings are composed each of seven distinct subunits, giving the 20S complex the general structure of α_1–7_β_1–7_β_1–7_α_1-7_. The catalytic sites are localized to some of the β subunits. Each extremity of the 20S barrel can be capped by a 19S RP each composed of 17 distinct subunits, 9 in a “base” subcomplex and 8 in a “lid” subcomplex. One important function of the 19S RP is to recognize ubiquitinated proteins and other potential substrates of the proteasome. Several ubiquitin-binding subunits of the 19S RP have been identified, although their biological roles and mode of action have not been discerned. A second function of the 19S RP is to open an orifice in the α ring that will allow entry of the substrate into the proteolytic chamber. Also, since a folded protein would not be able to fit through the narrow proteasomal channel, it was assumed that the 19S particle unfolds substrates and inserts them into the 20S CP. Both the channel opening function and the unfolding of the substrate require metabolic energy, and, indeed, the 19S RP “base” contains six different ATPase subunits. Following degradation of the substrate, short peptides derived from the substrate are released, as well as reusable ubiquitin (for a scheme describing the ubiquitin system, see Figure 5; for the structure of the 26S proteasome, see Figure 6).

## CONCLUDING REMARKS

The evolution of proteolysis as a centrally important regulatory mechanism has served as a remarkable example for the evolution of a novel biological concept and the accompanying battles to change paradigms. The five-decade journey between the early 1940s and early 1990s began with fierce discussions on whether cellular proteins are static, as has been thought for a long time, or are turning over. The discovery of the dynamic state of proteins was followed by the discovery of the lysosome that was believed—between the mid-1950s and mid-1970s—to be the organelle within which intracellular proteins are destroyed. Independent lines of experimental evidence gradually eroded the lysosomal hypothesis and resulted in a new idea that the regulated degradation of intracellular proteins under basal metabolic conditions was mediated by a non-lysosomal machinery. This resulted in the discovery of the ubiquitin system in the late 1970s and early 1980s. Interestingly, modifications of different target substrates by ubiquitin and ubiquitin-like proteins are now known to be involved in all aspects of lysosomal degradation, such as in the generation of the autophagic vacuoles, and in the routing of cargo-carrying vesicles to the lysosome (see below). Modifications by ubiquitin and ubiquitin-like proteins are now viewed, much like phosphorylation, as a mechanism to generate recognition elements *in trans* on target proteins to which downstream effectors bind. In one case, generation of Lys–48-based polyubiquitin chains, the binding effector is the 26S proteasome that degrades the ubiquitin-tagged protein. In many other cases, different modifications serve numerous proteolytic (lysosomal) and non-proteolytic functions, such as routing of proteins to their subcellular destinations. We were fortunate at the beginning of our studies to have in mind a clear distinction between lysosomal and non-lysosomal proteolytic systems, not knowing what we know nowadays that the two processes are linked to one another and are mediated via similar modifications. Had we known that, our route would have been much more complicated.

**Figure 7:**
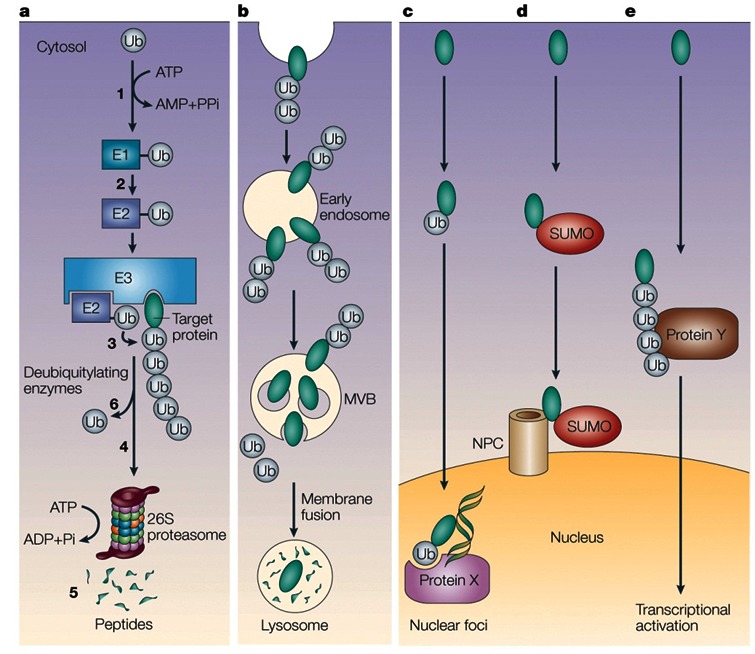
**Some of the different functions of modification by ubiquitin and ubiquitin-like proteins.** **a:** Proteasomal-dependent degradation of cellular proteins (see Figure 4). **b:** Mono- or oligoubiquitination targets membrane proteins to degradation in the lysosome/vacuole. **c:** Monoubiquitination, or **d:** a single modification by a ubiquitin-like (UBL) protein, SUMO for example, can target proteins to different subcellular destinations such as nuclear foci or the nuclear pore complex (NPC). Modification by UBLs can serve other, non-proteolytic, functions, such as protecting proteins from ubiquitination or activation of E3 complexes. **e:** Generation of a Lys–63-based polyubiquitin chain can activate transcriptional regulators, directly or indirectly (via recruitment of other proteins (protein Y; shown), or activation of upstream components such as kinases). Ub, ubiquitin; K, Lys; S, Cys (with permission from Nature Publishing Group; published originally in Ciechanover et al.^83^).

**Figure 8:**
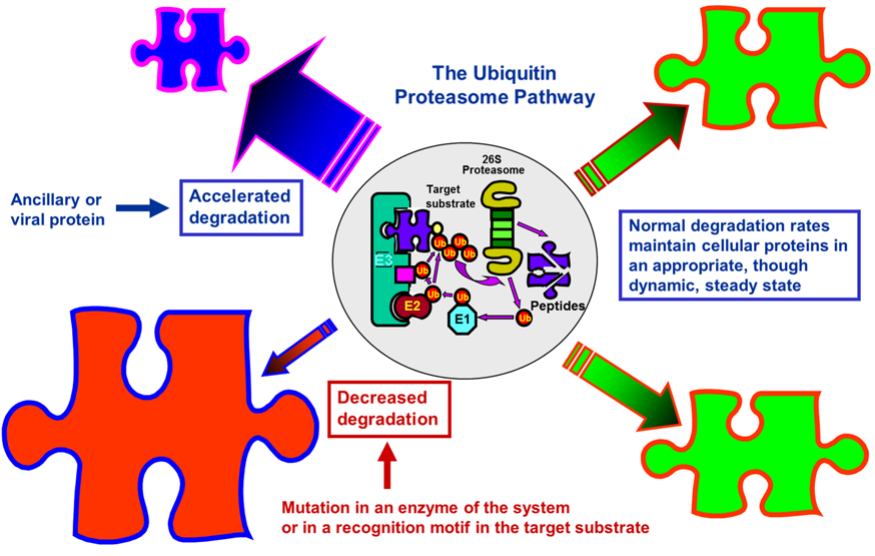
**Aberrations in the ubiquitin-proteasome system and pathogenesis of human diseases.** Normal degradation of cellular proteins maintains them in a steady-state level, though this level may change under various pathophysiological conditions (upper and lower right side). When degradation is accelerated due an increase in the level of an E3 (Skp2 in the case of p27, for example), or overexpression of an ancillary protein that generates a complex with the protein substrate and targets it for degradation (the human papillomavirus E6 oncoprotein that associates with p53 and targets it for degradation by the E6-AP ligase, or the cytomegalovirus-encoded ER proteins US2 and US11 that target MHC class I molecules for ERAD), the steady-state level of the protein decreases (upper left side). A mutation in a ubiquitin ligase (such as occurs in adenomatous polyposis coli (APC), or in E6-AP (Angelmans’ syndrome)) or in the substrate’s recognition motif (such as occurs in β-catenin or in ENaC) will result in decreased degradation and accumulation of the target substrate.

With the identification of the reactions and enzymes that are involved in the ubiquitin-proteasome cascade, a new era in the protein degradation field began at the late 1980s and early 1990s. Studies that showed that the system was involved in targeting of key regulatory proteins—such as light-regulated proteins in plants, transcriptional factors, cell cycle regulators, and tumor suppressors and promoters—started to emerge.^74^–^78^ They were followed by numerous studies on the underlying mechanisms involved in the degradation of specific proteins, each with its own unique mode of recognition and regulation. The unraveling of the human genome revealed the existence of hundreds of distinct E3s, attesting to the complexity and the high specificity and selectivity of the system. Two important advances in the field were the discovery of the non-proteolytic functions of ubiquitin, such as activation of transcription and routing of proteins to the vacuole, and the discovery of modification by ubiquitin-like proteins (UBLs) that are also involved in numerous non-proteolytic functions such as directing proteins to their subcellular destination, protecting proteins from ubiquitination, or controlling entire processes such as autophagy (see, for example, Mizushima et al.^79^) (for the different roles of modifications by ubiquitin and UBLs, see Figure 7). All these studies have led to the emerging realization that this novel mode of covalent conjugation plays a key role in regulating a broad array of cellular process—among them cell cycle and division, growth and differentiation, activation and silencing of transcription, apoptosis, the immune and inflammatory response, signal transduction, receptor-mediated endocytosis, various metabolic pathways, and the cell quality control—through proteolytic and non-proteolytic mechanisms. The discovery that ubiquitin modification plays a role in routing proteins to the lysosome/vacuole and that modification by specific and unique ubiquitin-like proteins and modification system controls autophagy closed an exciting historical cycle, since it demonstrated that the two apparently distinct systems communicate with one another. With the many processes and substrates targeted by the ubiquitin pathway, it has not been surprising to find that aberrations in the system underlie, directly or indirectly, the pathogenesis of many diseases. While inactivation of a major enzyme such as E1 was obviously lethal, mutations in enzymes or in recognition motifs in substrates that do not affect vital pathways, or that affect the involved process only partially, may result in a broad array of phenotypes. Likewise, acquired changes in the activity of the system can also evolve into certain pathologies. The pathological states associated with the ubiquitin system can be classified into two groups: 1) those that result from loss of function—mutation in a ubiquitin system enzyme or in the recognition motif in the target substrate that result in stabilization of certain proteins, and 2) those that result from gain of function—abnormal or accelerated degradation of the protein target (for aberrations in the ubiquitin system that result in disease states, see Figure 8). Studies that employ targeted inactivation of genes coding for specific ubiquitin system enzymes and substrates in animals can provide a more systematic view into the broad spectrum of pathologies that may result from aberrations in ubiquitin-mediated proteolysis. Better understanding of the processes and identification of the components involved in the degradation of key regulatory proteins will lead to the development of mechanism-based drugs that will target specifically only the involved proteins. While the first drug, a specific proteasome inhibitor, is already on the market,^80^ it appears that one important hallmark of the new era we are entering now will be the discovery of novel drugs based on targeting of specific processes such as inhibiting aberrant Mdm2- or E6-AP-mediated accelerated targeting of the tumor suppressor p53 which will lead to regain of its lost function.

Many reviews have been published on different aspects of the ubiquitin system. The purpose of this article is to bring to the reader several milestones along the historical pathway which led to the discovery of the ubiquitin system. For additional reading on the ubiquitin system, the reader is referred to numerous review articles written on the subject (for some older reviews, see for example Glickman et al.^81^ and Pickart et al.^82^). Some parts of this review, including several figures, are based on another published review article.^83^

## Abbreviations:

APF-1: ATP-dependent proteolysis factor 1 (ubiquitin);
CP: 20S core particle (of the proteasome);
G6PD: glucose-6-phosphate dehydrogenase;
MCP: multicatalytic proteinase complex (26S proteasome);
ODC: ornithine decarboxylase;
PEPCK: phosphoenol-pyruvate carboxykinase;
RP: 19S regulatory particle (of the proteasome);
TAT: tyrosine aminotransferase;
UBIP: ubiquitous immunopoietic polypeptide (ubiquitin).
